# Safe and Orally Bioavailable Inhibitor of Serine Palmitoyltransferase Improves Age-Related Sarcopenia

**DOI:** 10.1021/acsptsci.4c00587

**Published:** 2024-12-29

**Authors:** Johanne Poisson, Ioanna Daskalaki, Vijay Potluri, Jean-David Morel, Sandra Rodriguez-Lopez, Alessia De Masi, Giorgia Benegiamo, Suresh Jain, Tanes Lima, Johan Auwerx

**Affiliations:** † Laboratory of Integrative Systems Physiology, École Polytechnique Fédérale de Lausanne (EPFL), Lausanne 1015, Switzerland; ‡ Intonation Research Laboratories, Hyderabad 500076, India

**Keywords:** inclusion body myositis, proteostasis, very-long chain ceramides, deoxy-sphingolipids, amyloid beta

## Abstract

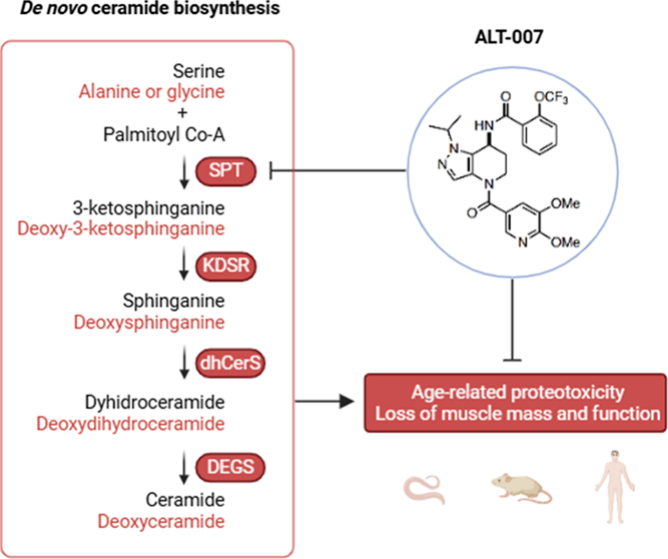

The accumulation of ceramides and related metabolites has emerged as a pivotal mechanism contributing to the onset of age-related diseases. However, small molecule inhibitors targeting the ceramide *de novo* synthesis pathway for clinical use are currently unavailable. We synthesized a safe and orally bioavailable inhibitor, termed ALT-007, targeting the rate-limiting enzyme of ceramide *de novo* synthesis, serine palmitoyltransferase (SPT). In a mouse model of age-related sarcopenia, ALT-007, administered through the diet, effectively restored muscle mass and function compromised by aging. Mechanistic studies revealed that ALT-007 enhances protein homeostasis in *Caenorhabditis elegans* and mouse models of aging and age-related diseases, such as sarcopenia and inclusion body myositis (IBM); this effect is mediated by a specific reduction in very-long chain 1-deoxy-sphingolipid species, which accumulate in both muscle and brain tissues of aged mice and in muscle cells from IBM patients. These findings unveil a promising therapeutic avenue for developing safe ceramide inhibitors to address age-related neuromuscular diseases.

## Introduction

Bioactive sphingolipids constitute a class of lipids with diverse cellular functions, such as regulation of energy metabolism, apoptosis, senescence and inflammatory responses.^1^ The shingolipidome is highly complex with distinct molecular species and pathways that dictate the specific signaling functions of sphingolipids. Ceramides are the fundamental building blocks of all complex sphingolipids and their accumulation has been implicated in numerous age-related conditions including neurodegeneration, inflammation, cancer, metabolic diseases, and cardiovascular disorders.^2,3^ Despite the well-established implication of ceramides in age-related disorders, there are currently no approved pharmacological approaches to target ceramide *de novo* synthesis for addressing these conditions.

The enzymatic pathways orchestrating the biosynthesis, degradation, and regeneration of ceramides play a pivotal role in shaping the composition of the cellular sphingolipidome.^2^ Ceramide *de novo* biosynthesis initiates with the condensation of palmitoyl-CoA and serine by serine palmitoyltransferase (SPT). The byproduct of this reaction, 3-ketosphinganine, is converted to sphinganine by 3-ketosphinganine reductase (KDSR) and a family of six ceramide synthases (CERS1–6) catalyzes n-acylation reactions on sphinganine, producing diverse dihydroceramides with acyl chain lengths ranging from 14 to 34 carbon atoms. In the last chemical step, dihydroceramide desaturases (DES1 and DES2) introduce a 4,5-trans-double bond, yielding ceramides, which serve as a scaffold for complex sphingolipids. Ceramides can then be either stored or recycled through sphingomyelin hydrolysis or salvage pathways. Under certain conditions, such as variations in the alanine/serine ratio and missense mutations in SPT subunits, SPT can use alternative amino acids, particularly l-alanine and glycine, resulting in the production of 1-deoxysphingolipids (1-deoxySLs).^4,5^ These species exhibit structural deviations from typical sphingolipids, characterized by the absence of essential C1–OH groups, rendering them unable to be degraded by canonical catabolic pathways. Elevated levels of 1-deoxySLs are involved in several neurological and metabolic disorders.^4,6^ The most effective strategy to deplete ceramides and their associated toxic metabolites is to inhibit the rate-limiting enzyme in their *de novo* synthesis, SPT. Myriocin is the foremost extensively studied SPT inhibitor in preclinical settings. It was discovered in a quest to identify the active component of *Isaria Siclairii*, a fungus commonly used in traditional Chinese medicine.^7^ Myriocin is also enriched in related fungal species such as the Cordyceps.^8^ Although myriocin was found to be toxic and did not progress to clinical trials, drug discovery efforts were initiated to develop less toxic myriocin-like compounds. In one successful campaign, extensive chemical modification of the myriocin scaffold, including removal of side chain functionalities and elimination of chiral centers, led to a novel potent immunosuppressant, fingolimod, also known as FTY720, which was approved for clinical use in multiple sclerosis.^9^ Novel biaryl acid chemotypes have been identified as SPT inhibitors and subsequent optimization in the design and synthesis has yielded two hits with suitable in vitro and pharmacokinetic (PK) profiles.^10^ However, these molecules exhibit toxicity after prolonged exposure in rats. Through an alternative method involving a high-throughput screening of in-house compound library and subsequent medicinal chemistry optimization, another group developed a safer bioavailable SPT inhibitor which displayed a suitable PK profile, no toxicity, and potential for use as a cancer treatment.^11^

Previous findings using myriocin underscored the efficacy of depleting ceramides in mitigating muscle deterioration in mouse models of Duchenne muscular dystrophy (DMD) and age-related sarcopenia.^12,13^ Further, mechanistic studies unveiled that ceramide depletion contributes to enhanced muscle function by improving protein homeostasis.^14^ Leveraging existing structures of novel SPT inhibitors, we synthesized these compounds and evaluated their potential to reduce protein aggregates.^14^ One particularly promising compound which was found to be safe and bioavailable,^11^ here code-named ALT-007, exhibited great potency in depleting ceramides and clearing protein aggregates in primary muscle cells obtained from inclusion body myositis (IBM) patients and aged donors.^11^

In this study, we evaluated the efficacy of ALT-007 in a mouse model of age-related sarcopenia. Our investigations demonstrate the therapeutic potential of ALT-007 to mitigate age-related muscle loss and fitness decline potentially by reducing the levels of deoxy-sphingolipids of very-long chains and their impact on cellular protein homeostasis. Our findings highlight ALT-007 as a safe and orally bioavailable compound with potential to mitigate neuromuscular diseases.

## Results

### ALT-007 is Safe, Orally Bioavailable and Reduces Ceramides in Skeletal Muscle

**Figure 1:**
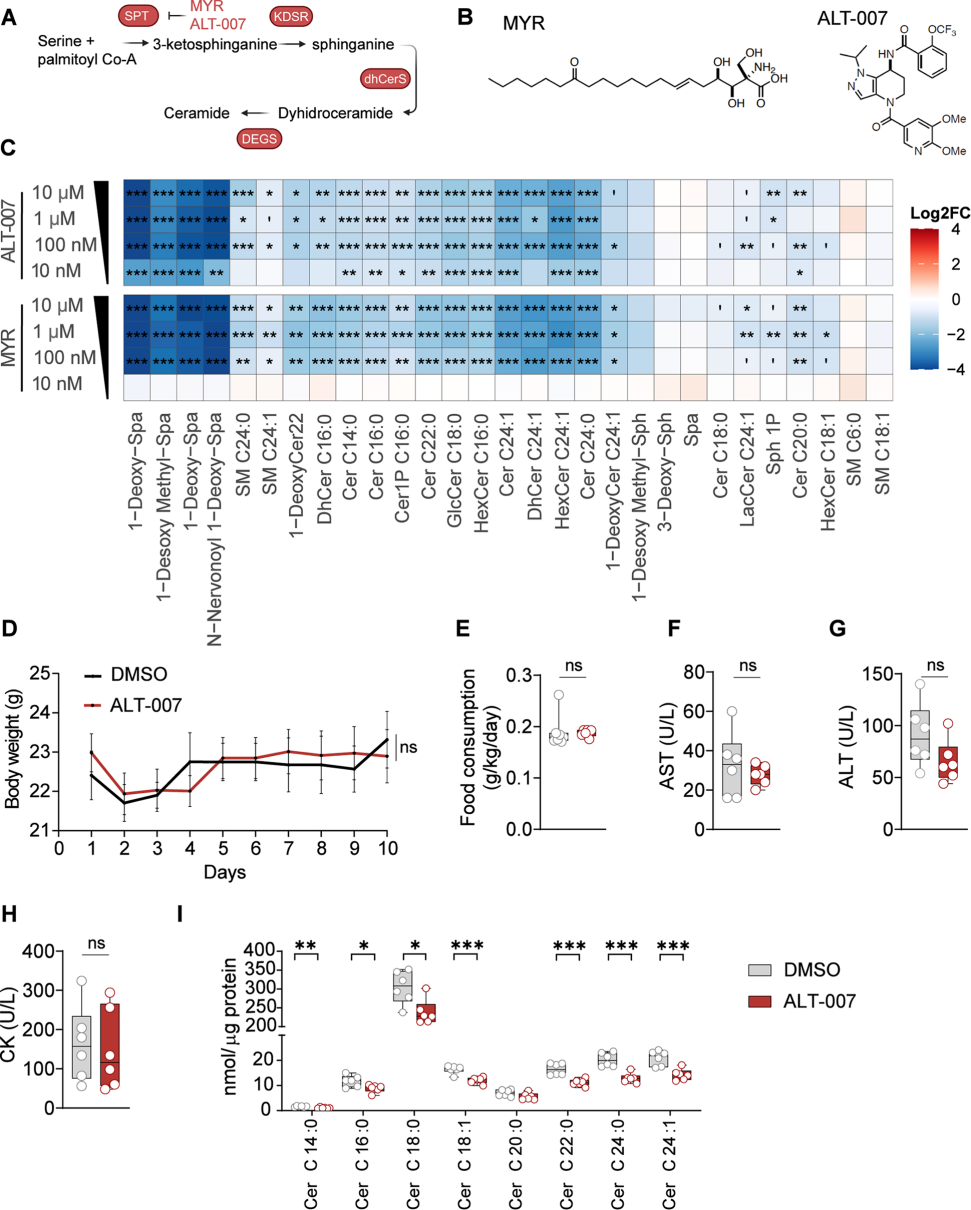
ALT-007, an orally bioavailable SPT inhibitor, is more potent than myriocin, safe and effective in the skeletal muscle. (A) Schematic representation of the *de novo* sphingolipid synthesis pathway. (B) Chemical structures of myriocin (MYR) and ALT-007. (C) Heatmap analysis comparing the effects of treatments with the indicated concentrations of MYR and ALT-007 on lipid species in C2C12 cells. *N* = 3 per group. Data are shown as log_2_ fold change. (D) Body weight curves of mice treated with ALT-007 or vehicle (4 mg/kg/d for 10 days, in the food). (E) Mean food consumption measurement throughout the treatment. (F) Plasma levels of aspartate transaminase (AST), (G) alanine transaminase (ALT) and (H) creatine kinase (CK) after 10 days of treatment with ALT-007 at 4 mg/kg/day. (I) Ceramide levels measured in the gastrocnemius muscle after 10 days of treatment. *N* = 8 per group. Quantitative data are expressed as median with min to max values. *P < 0.05, **P < 0.01, ***P < 0.001. ANOVA test was used for multiple comparison, and the Mann–Whitney *U* test when comparing 2 groups. All tests were 2 sided. Abbreviation: SPT, serine palmitoyltransferase; CK, creatine kinase; MYR, Myriocin; ns, nonsignificant.

In this study, we synthesized and tested ALT-007, an SPT inhibitor (Figure 1A) previously shown to be safe and orally bioavailable.^11^ The differences in the central core ring system of ALT-007, compared to Myriocin (Figure 1B), confers the molecule improved metabolic stability, aqueous solubility and permeability.^11^ We confirmed the efficacy of ALT-007 in lowering ceramide species with superior potency compared to Myriocin in immortalized mouse C2C12 myoblasts as shown by its effect to lower ceramide levels at a concentration of 10 nM (Figure 1C). The favorable pharmacokinetic profile shown previously in mice^11^ and target engagement in vivo was also confirmed (Figure 1C,I). Mice treated with ALT-007 via food admix showed no differences in body weight and food intake compared to DMSO control mice after 10 days of treatment (Figure 1D,E). Furthermore, neither hepatic nor muscle toxicity was identified by plasma measurements of transaminase and creatine kinase (CK) levels (Figure 1F–H). Moreover, ALT-007 effectively decreased ceramide levels with a stronger effect on very long-chain (VLC) species in the gastrocnemius muscle (Figure 1I).

## ALT-007 is Well Tolerated in Long-Term Oral Administration and Improves Overall Fitness in Aging

**Figure 2:**
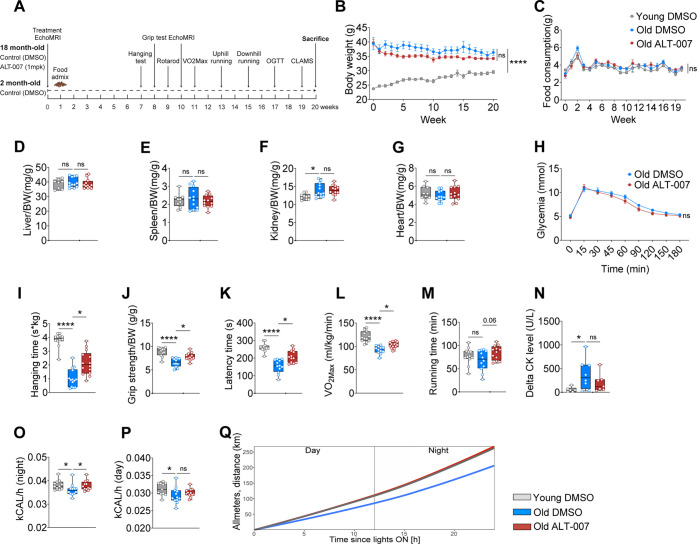
Chronic oral administration of ALT-007 enhances physical fitness in aged mice. (A) Schematic representation of the study time-line. Compounds were mixed in the food, ALT-007 at 1 mg/kg and DMSO at equivalent volume. At week 0: young mice were 2 months old, and the old mice were 18 months old. (B) Body weight curve over the course of the study (20 weeks). (C) Food intake curve throughout the study. (D–G) Organ mass at sacrifice (week 20), normalized to body weight. (H) Oral glucose tolerance test (oGTT) performed at week 17. (I) Best performance during hanging time normalized to BW at week 7. (J) Best performance during grip strength on the 4 limbs normalized to BW at week 8. (K) Rotarod’s best latency time at week 9. (L) VO_2Max_ performed at week 11. (M) Uphill maximum running time performed at week 13. *N* = 9 per group. (N) Difference of CK level after—before 90 min downhill running performed at week 15. *N* = 6 in young, *N* = 8 in DMSO and *N* = 7 in ALT-007-treated groups. (O–Q) Measurement of kCAL/h at night (O) and at day (P) and movement recording in *x*, *y*, *z* (Q) during 24 h in CLAMS at week 19. *N* = 12 per group unless specified otherwise. Quantitative data are expressed as median with IQR. *P < 0.05, **P < 0.01, ***P < 0.001, ****P < 0.0001. ANOVA test was used for multiple comparison, and the Mann–Whitney *U* test when comparing 2 groups. All tests were 2 sided.

We next aimed to test whether ALT-007 given orally could mitigate age-related sarcopenia in mice. Aged C57BL/6J mice (18–32 months old) are widely used to model age-related loss of muscle mass and function, termed sarcopenia, as they replicate the biochemical and physiological features of muscle aging in humans, providing an ideal model for testing therapeutic interventions.^13,15^ Aged 18 month mice were treated with either ALT-007 (1 mg/kg) or DMSO mixed in the food. A group of young, 2-month-old mice, were treated with DMSO mixed in the food as another control group. The intervention in all groups lasted for 20 weeks in total. After 7 weeks of treatment, in vivo muscle performance and overall fitness tests were performed (Figure 2A). In agreement with our pharmacodynamic experiment (Figure 1C–I), chronic treatment with ALT-007 was well tolerated, as indicated by stable body weight and absence of changes in food intake (Figure 2B,C) and in tissue weights relative to control (Figure 2D–G). Furthermore, ALT-007-treated mice displayed no gastrointestinal (GI) toxicity as indicated by the absence of a difference in plasma citrulline levels compared to untreated mice^16,17^ (Supporting Information Figure 1A). SPT inhibition has been shown to improve insulin sensitivity in obesity models;^3^ in the aged mice in this study, we observed no difference in the glucose tolerance test with ALT-007 (Figure 2H).

Confirming proof-of-concept studies with Myriocin,^12^ muscle strength was significantly improved by ALT-007, as shown by maximum hanging time and grip strength, both of which were increased compared to aged control mice after 7 and 8 weeks of treatment with ALT-007 (Figure 2I,J). Similarly, coordination measured by the rotarod test was improved in aged mice after 9 weeks of treatment with ALT-007 (Figure 2K). VO_2max_, a critical marker of aerobic capacity and overall fitness, was also significantly higher in ALT-007 mice (Figure 2L). ALT-007 did not significantly improve the ability of aged mice to run uphill, nor did it reduce the levels of creatine kinase in the blood after a downhill running test, although there was a trend for improvement in both tests (Figure 2M,N). To evaluate energy expenditure and behavioral activity after 19 weeks of treatment, mice were placed in Comprehensive Lab Animal Monitoring System (CLAMS) for 2 days. ALT-007-treated mice displayed a significant increase in energy expenditure (measured by kCal/h) at night, their active period (Figure 2O,P). This is linked with increased activity, as ALT-007 completely restored the decline in global physical activity in aged mice compared to aged control counterparts (Figure 2Q). Interestingly, ALT-007-treated mice displayed activity levels comparable to young mice. These findings suggest that ALT-007 is well tolerated after long-term oral administration and attenuates the age-related decline in fitness.

## ALT-007 Attenuates Muscle Aging and Rewires Transcriptional Pathways

**Figure 3:**
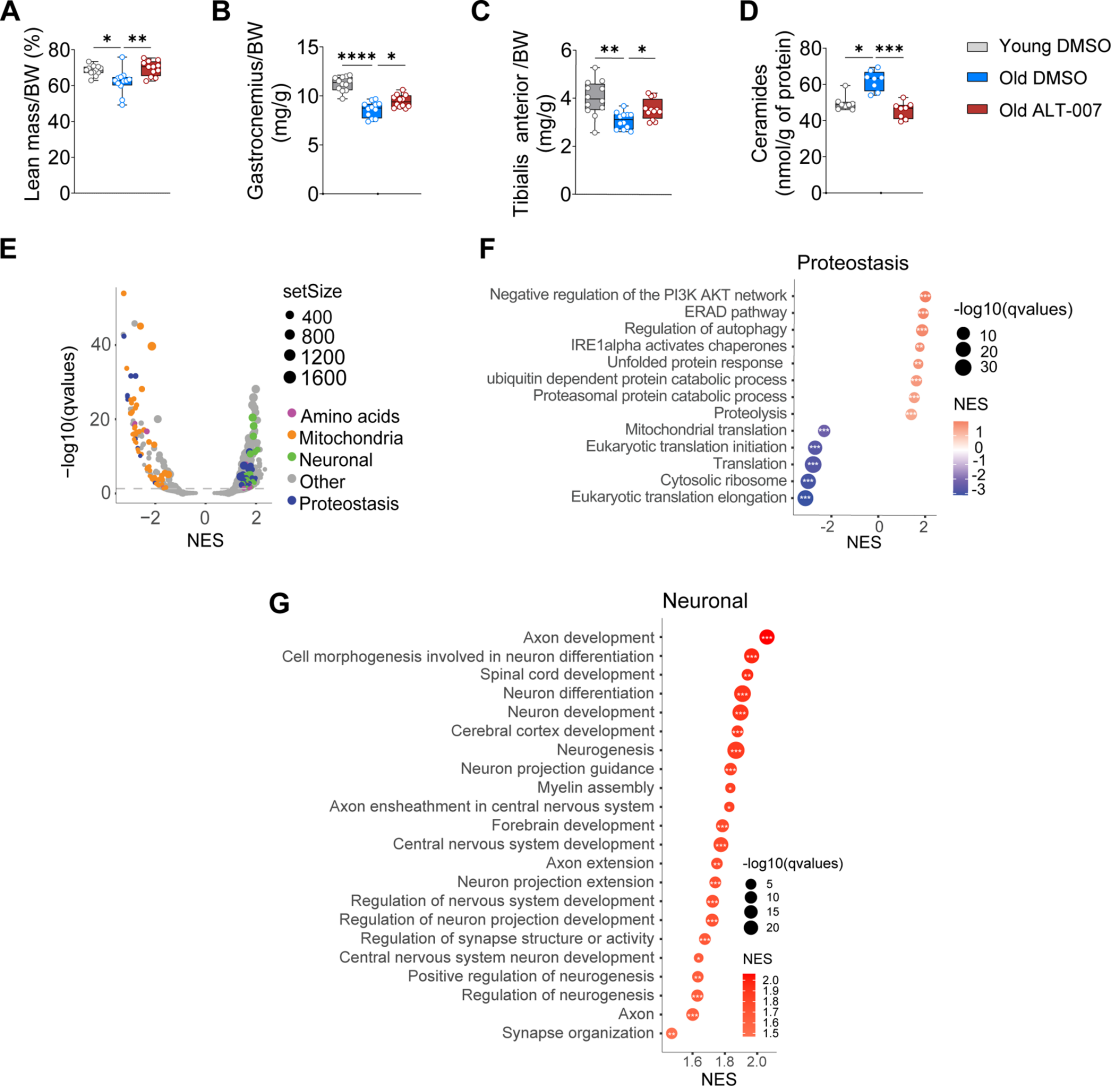
ALT-007 preserves muscle mass and rewires skeletal muscle gene expression during aging. (A) Lean mass measured by echoMRI after 11 weeks of treatment (ALT-007:1 mg/kg; DMSO: equivalent volume, for 20 weeks). (B) Gastrocnemius and (C) Tibialis anterior muscle weight after 20 weeks of treatment. *N* = 12 per group. (D) Total ceramide levels measured in the gastrocnemius muscle. (E) Volcano plot of mitochondrial, proteostasis, amino acids, biosynthetic processes, and neuronal-related gene sets highlighted. (F,G) Gene set enrichment analysis (GSEA) of (F) pathways involved in protein folding and degradation and (G) neurogenesis pathways in the skeletal muscle of mice treated with ALT-007 (*N* = 5 in DMSO and *N* = 4 in ALT-007-treated groups). Adjusted P values in GSEA comparison are indicated as follows: *adjusted *P* < 0.05; **adjusted *P* < 0.01; ***adjusted *P* < 0.001.

Mice treated with ALT-007 displayed increased lean mass measured by echoMRI after 11 weeks of treatment, suggesting elevated muscle mass (Figure 3A). In agreement, ALT-007 increased muscle weight in aged mice measured at sacrifice after 20 weeks of treatment (Figure 3B–C). The amount of ALT-007 administered in vivo was sufficient to inhibit SPT in the muscle as the gastrocnemius of ALT-007-treated mice displayed lower levels of total ceramides (Figure 3D) suggesting suitable target engagement.

To investigate the underlying mechanisms involved in the therapeutic effects of ALT-007, we performed RNaseq analysis in gastrocnemius muscles of aged mice treated with ALT-007 or DMSO. Gene set enrichment analysis (GSEA) revealed that ALT-007 impacted several age-related gene sets such as mitochondrial and proteostasis pathways, amino acids and biosynthetic processes, and neuronal-related gene sets (Figure 3E).

We have previously shown that ceramide depletion restores muscle function in aging by reducing protein aggregates in the muscle.^14^ In agreement, we observed enrichment of several proteostasis pathways involved in protein folding and degradation upon ALT-007 treatment including autophagy, unfolded protein response, and ubiquitin-dependent protein catabolic processes (Figure 3F). Conversely, ribosomal and translation pathways were significantly attenuated by ALT-007 (Figure 3F). These alterations may potentially converge to improve the clearance of protein aggregates as observed in vitro in primary myoblasts from both aged individuals and IBM patients treated with ALT-007.^14^

It has been reported that neurogenesis in muscle declines with age and that alteration in nerve-muscle communication is a contributing factor to the progression of age-related sarcopenia.^18^ Consistently, we found a remarkable upregulation of neurogenesis pathways in the muscle of ALT-007-treated mice (Figure 3G), suggesting that ALT-007 may be also impacting the peripheral nervous system that is innervating the muscle. In particular, GSEA analysis using the MsigDB mouse cell type signature sets verified overrepresentation of specific neuronal cell types in old treated mice compared to their control untreated counterparts. In addition, this was opposite to what we observed when comparing old versus young untreated mice (Supporting Information Figure 2).

## Role of 1-Deoxy-sphingolipids in Protein Aggregation

**Figure 4:**
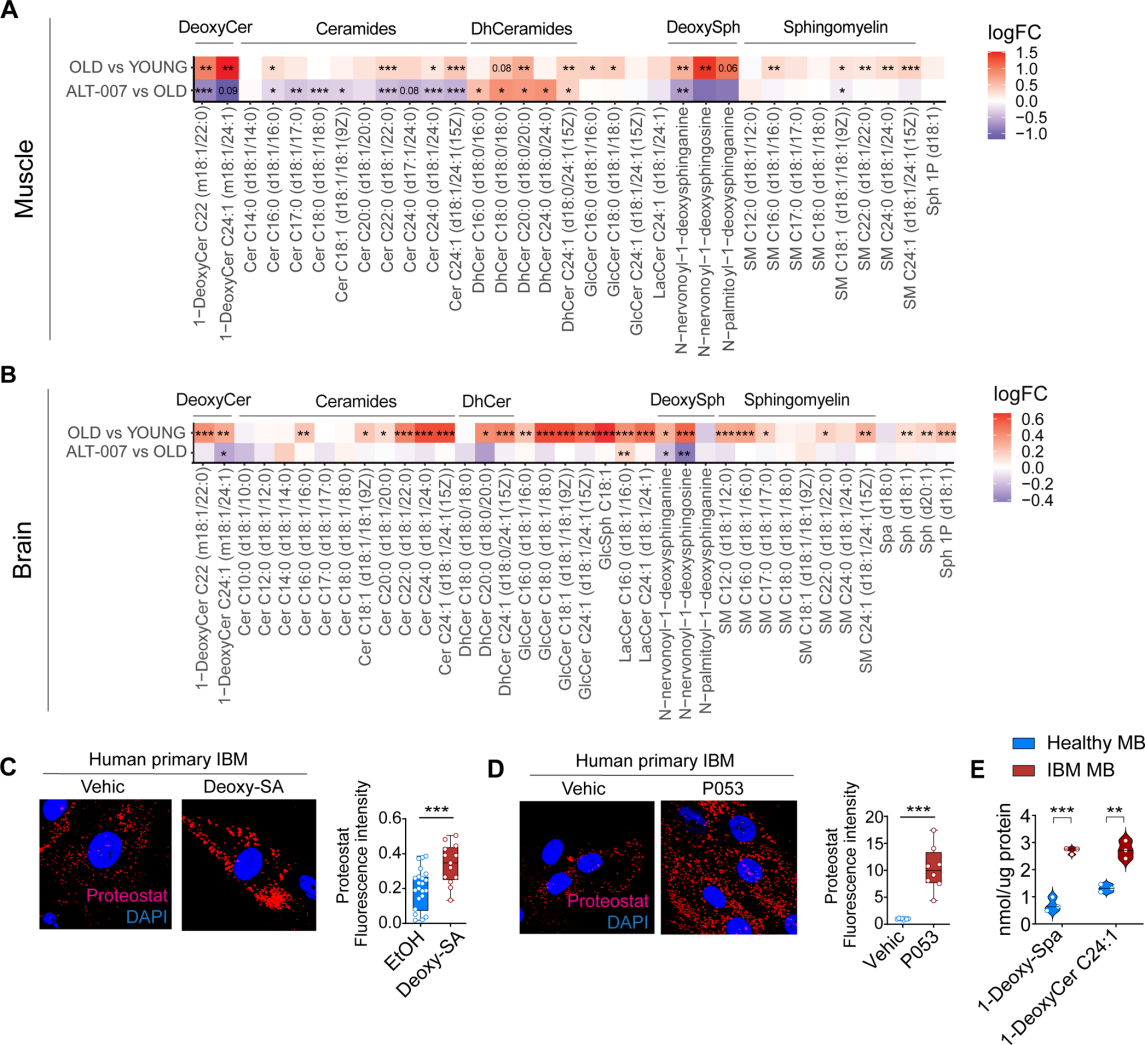
1-deoxy-SL species accumulate in muscle and brain tissues during aging and contribute to cellular protein aggregation. (A,B) Heat map representing the abundance of sphingolipids in (A) gastrocnemius muscle and (B) brain with and without treatment with ALT-007 in mice (ALT-007 at 1 mg/kg; DMSO at equivalent volume for 20 weeks). *N* = 8 per group. Data are shown as log_2_ fold change. Adjusted *P* values are indicated as follows: *adjusted *P* < 0.05; **adjusted *P* < 0.01; ***adjusted *P* < 0.001. (C,D) Representative images and quantification of protein aggregates upon 1-deoxy-SA (C) and P053 treatment (D) in human primary IBM cells; both were administered at 4 μM for 24 h. *N* = 8 per group. (E) Analysis of 1-Deoxy-Spa and 1-DeoxyCer 24:1 levels in myoblasts from healthy and IBM patients. *N* = 3 per group. (C–E) Quantitative data are expressed as median with IQR and compared using the Mann–Whitney *U* test. All tests were 2 sided. *P < 0.05, **P < 0.01, ***P < 0.001.

We performed a comprehensive sphingolipidome analysis aiming to identify specific sphingolipid species associated with ALT-007 effects in the muscle. 1-Deoxy-SLs of very long chain, particularly 1-deoxyCer 22, 1-deoxyCer 24:1 and the sphingoid base N–C24:1-deoxysphinganine, were the main sphingolipid species attenuated by ALT-007 in the gastrocnemius muscle (Figure 4A). The intracellular ratio of alanine and serine affects the production of 1-deoxy-SLs levels as these species are produced when SPT condensates alanine instead of serine. However, we did not detect differences in the alanine/serine ratio in the muscle of aged compared to young mice (Supporting Information Figure 1B), suggesting that age-related changes in deoxy-SLs may be the result of other mechanisms.^4^ Interestingly, ALT-007 also attenuated the age-related increase in 1-deoxy-SLs in the brain (Figure 4B) suggesting a potential effect of ALT-007 beyond skeletal muscle in the central nervous system.^19^

Our data (Figure 4) suggest that very-long-chain (VLC) 1-deoxy-SLs may contribute to age-related sarcopenia. Based on previous findings^14^ we reasoned that this could be mechanistically linked with the decline in proteostasis. To assess the cell-autonomous impact of deoxy-SLs on proteostasis, we enhanced the production of VLC 1-deoxy-SLs in primary myoblasts derived from IBM patients, and subsequently measured protein aggregation. In the first experiment, we treated human IBM cells with 1-deoxy-sphinganine (1-deoxy-SA), a precursor of VLC 1-deoxy-SLs. The second approach involved shifting the production of long to very-long sphingolipid species using P053, a potent high-affinity inhibitor of CerS1. As we have previously shown, *Cers1* gene silencing increases *Cers2* in mouse myoblasts, while concomitant knockdown of both *Cers1* and *Cers2* was sufficient to significantly ameliorate impaired differentiation potential.^20^ These findings suggest that *Cers1* inhibition predominantly triggers *Cers2* elevation, the second most abundant *CerS* enzyme in the muscle after *CerS1*,^21^ to produce sphingolipid species with acyl chains of 22–24 carbons length, including 1-deoxy-SLs. Our findings revealed that both 1-deoxy-SA and P053 treatment significantly exacerbated the accumulation of cellular protein aggregates in IBM cells, as evidenced by increased proteostat signal (Figure 4C,D). Consistent with these observations, human myoblasts from IBM patients exhibited elevated levels of 1-deoxy-SA and 1-deoxy-Cer 24:1 compared to myoblasts from healthy subjects (Figure 4E), which correlated with heightened protein aggregation.^14^ These results suggest that VLC deoxy-SLs may act as potential disruptors of protein homeostasis in skeletal muscle, a phenomenon that can be potentially mitigated through oral treatment with ALT-007.

## ALT-007 Improves Proteotoxicity and Increases Lifespan in *Caenorhabditis elegans*

**Figure 5:**
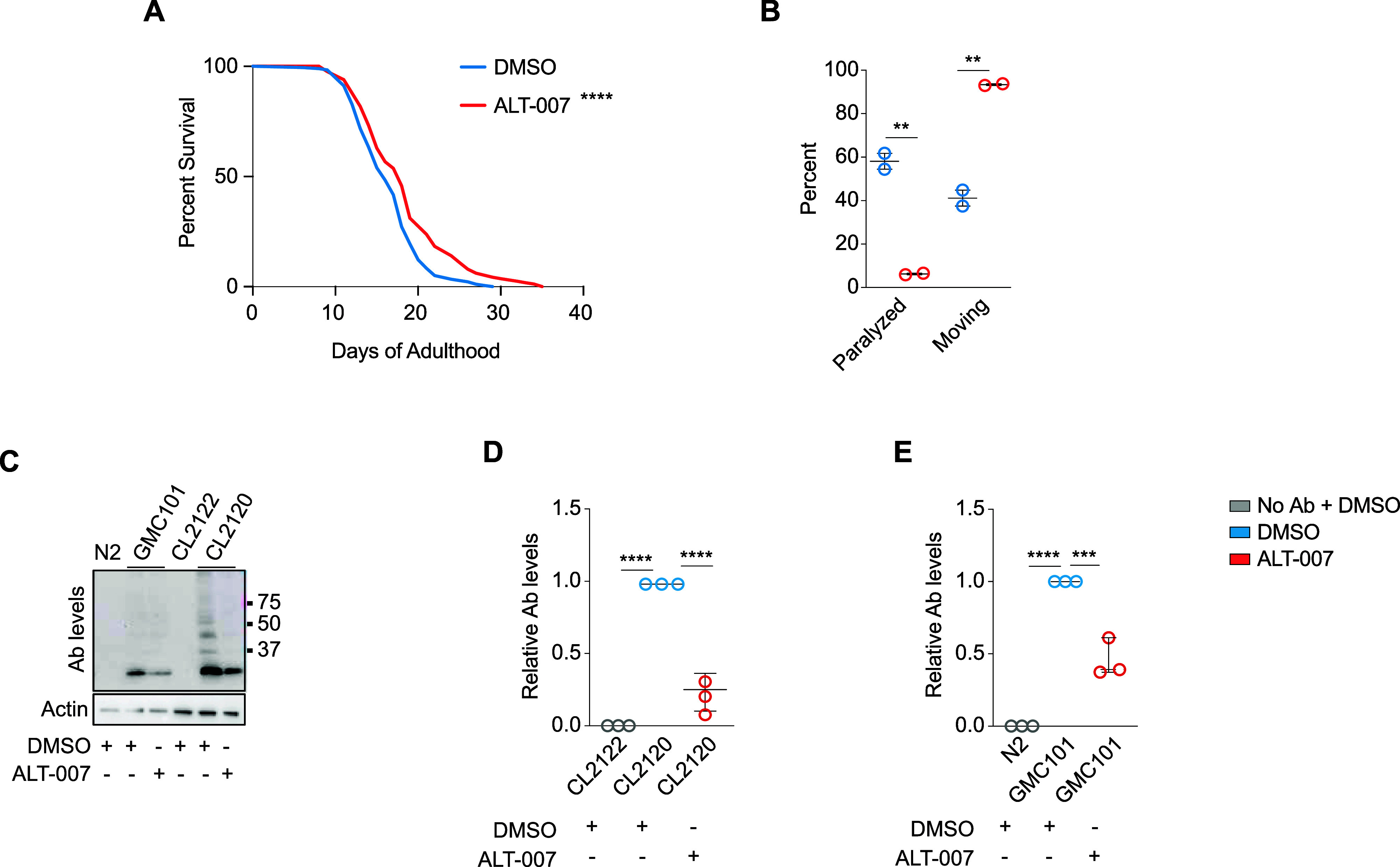
ALT-007 increases lifespan, improves healthspan and reduces protein aggregation in *C. elegans*. (A) Median and maximal lifespan of N2 wild-type*C. elegans* animals treated with ALT-007 or vehicle (5 μM). *N* = 2 biological replicates with at least 170 animals per group. (B) Paralysis and movement analysis of amyloid beta (Ab)-expressing animals treated with ALT-007 or vehicle (1 μM) at day 1 of adulthood. *N* = 2 biological replicates with at least 50 animals per group. (C) Amyloid aggregation upon ALT-007 treatment and DMSO as a control in GMC101 and CL2120 amyloid beta expressing animals and their respective controls. *N* = 3 biological replicates with at least 100 animals per group. (D) Quantification of amyloid beta (Ab) levels upon ALT-007 treatment. The log-rank (Mantel–Cox) test was used to evaluate differences between survival curves as indicated in Supplementary Table 1. two-tailed *t*-test was performed for two-group analysis and one-way analysis of variance (ANOVA) for three-group analysis.

To test whether ALT-007 has conserved beneficial effects across taxa, and given that the ceramide *de novo* biosynthesis pathway is conserved in *C. elegans*, we administered ALT-007 to the nematode*C. elegans*. To examine whether ceramides play a role in age-related decline in the worm, as observed in mammals, we first tested its effect lifespan. We found that treatment with ALT-007 significantly increased*C. elegans* median and maximal lifespan (Figure 5A and Supporting Information Table 1). Moreover, administration at the embryo stage, was nontoxic for animal development, as animals grew normally. Further, to test whether ALT-007 affects proteostasis, we treated GMC101 worms, which express the human amyloid beta isoform 1–42 in muscle cells, and assessed their locomotion capacity. Expression of amyloid beta in the worm muscle, leads to a severe paralysis phenotype within a few hours under triggering conditions. In agreement with data in mice and human cells, we found that ALT-007 treatment can reduce paralysis induced by amyloid beta aggregation by reducing amyloid beta aggregates in*C. elegans* (Figure 5B,C). The capacity of ALT-007 to reduce aggregation was further confirmed using an additional*C. elegans* strain that expresses amyloid beta aggregates under inducing conditions (CL2120 and CL2122 as a control) (Figure 5C,D). These findings suggest that ALT-007 ameliorates age-related decline by rewiring proteostasis across species.

Sphingolipids have emerged as important signaling molecules in health and disease. Recent findings in preclinical models have suggested that pharmacological approaches to modulate the ceramide *de novo* synthesis pathway can be an effective approach for treating various forms of neuromuscular degenerative diseases.^2,3,19^ SPT has been viewed as an attractive target for drug discovery, given the numerous studies showing that blocking ceramide biosynthesis counteracts disease phenotypes in a wide range of animal models. These studies have mostly used myriocin, which is a potent immunosuppressor derived from a natural product^7^ and which has not been advanced into clinical trials given its potential GI toxicity. The development of synthetic SPT inhibitors is therefore the scope of current drug development efforts. Previous work has characterized potent SPT inhibitors with enzymatic, binding, kinetic, and cellular assays.^10,11^ ALT-007 was shown to have a suitable pharmacological profile, is orally bioavailable and non-toxic in long-term dosing in rodents.^11^ We have previously shown that this compound has a potent effect in reducing protein aggregates in C2C12 myoblasts carrying the APP_swe_ mutation and in human myoblasts from aged donors and patients with IBM.^14^ Here we revealed that ALT-007, given orally, displays good pharmacodynamic properties in mice as indicated by a lack of toxicity in different tissues, maintenance of body weight, and reduction of several ceramide species in the gastrocnemius muscle after 10 days of treatment by food admix. Furthermore, we found that ALT-007 attenuates age-related muscle and physical decline in mice, potentially by reducing VLC-1-deoxy SLs and rewiring proteostasis pathways.

Notably, ALT-007 was able to restore muscle function, coordination, and aerobic capacity in aged mice which also maintained their muscle mass. We also found that ALT-007 fed to*C. elegans* is nontoxic and can significantly extend median and maximal lifespan, indicating that its mechanism of action to delay age-dependent deterioration is conserved across species. These effects confirm the potential therapeutic efficacy of inhibiting ceramide *de novo* synthesis in aging as previously shown with myriocin via IP injections and by genetically depleting SPT through AAV-mediated gene silencing of SPTLC1.^13,14^ Taken together, these findings suggest that inhibiting SPT via oral administration of ALT-007 has on-target effects on attenuating the skeletal muscle and general physiological decline caused by aging.

To better understand the molecular changes associated with the ALT-007 effects, we performed RNAseq. Gene Set Enrichment Analysis (GSEA) showed that pathways associated with the regulation of RNA metabolism and lular biosynthetic processes, neurogenesis, and protein homeostasis pathways were enriched in the muscle of treated mice. The induction of proteostasis pathways such as protein folding, proteolysis and autophagy by ALT-007 is pronounced and aligned with previous data showing that inhibition of ceramide synthesis reduces protein aggregation in vitro and in vivo.^14^ Consistent with these observations, we found that ALT-007 could ameliorate paralysis triggered by the toxic accumulation of amyloid beta aggregates in *C. elegans* body wall muscle cells.

In order to elucidate the downstream metabolites underlying the beneficial effects of ALT-007, we conducted a thorough targeted sphingolipidomic analysis in muscle tissue. Our findings revealed that ALT-007 mitigated the age-related increase in deoxy SLs, particularly targeting very long-chain species such as 1-DeoxyCer C22, 1-DeoxyCer C24:1, and *N*-nervonoyl-1-deoxysphinganine. To demonstrate the detrimental cell-autonomous effects of 1-deoxy SLs in muscle cells, we treated primary myoblasts from IBM patients with 1-deoxy SA or a CerS1 inhibitor, which augments the levels of very long-chain deoxy SLs; both strategies to increase deoxy-SLs exacerbated protein aggregation. These findings underscore an underappreciated link between 1-deoxy SLs and age-related protein aggregation. Consistently, 1-Deoxy SA and 1-DeoxyCer C24:1 species were found elevated in primary myoblasts from IBM patients compared to healthy age-matched individuals.

Age-related impairment in motor performance is not only due to defects inherent to the muscle, but also to loss of motor units, axonal atrophy, demyelination, and alterations in the electrical signal transduction in the neuromuscular junction.^22^ ALT-007 upregulated several pathways involved in neuron health in muscle bulk RNASeq data, potentially contributing to the better motor coordination found in treated mice. Moreover, cell decomposition analysis indicated that the muscles of old ALT-007-treated mice contained more neuronal cells than the untreated controls. It will be important in the future to test whether age-related alterations in the peripheral neurons can be caused by dysregulation of 1-deoxy SLs and whether ALT-007 can attenuate these alterations. In agreement, accumulation of 1-deoxy SLs have been implicated in various neuropathies,^4^ suggesting that ALT-007 might activate neuronal pathways and improve coordination in aging by mitigating the accumulation of 1-deoxy SLs. Interestingly, a parallel reduction in 1-deoxy SLs species was also observed in the brain upon systemic ALT-007 treatment, suggesting the compound’s ability to penetrate the blood–brain barrier.

In summary, our findings suggest that ALT-007 is a promising tool compound which supports the notion to develop safer, orally available serine palmitoyltransferase (SPT) inhibitors to treat neuromuscular diseases. Given their broad mode of action, involving beneficial effects on ceramide homeostasis, mitochondrial function, and proteostasis, such SPT inhibitors may offer significant therapeutic benefits in various inherited (facioscapulohumeral, oculopharyngeal, and Duchenne muscular dystrophies) as well as acquired (sarcopenia, disuse- and ICU-related myopathy,···) muscle disorders.^12−14^ Neuromuscular diseases (amyotrophic lateral sclerosis, spinal muscular atrophy, hereditary spastic paraplegia) and neurodegenerative conditions (Alzheimer’s, Parkinson’s, and Huntington’s Disease) are also attractive therapeutic targets for SPT inhibition, given that some of these diseases have been associated with abnormal ceramide levels^23,24^ and altered SPT activity.^25,26^ Furthermore, our data show that SPT inhibition increases the expression of markers of neuronal health and reduces brain and muscle deoxy-SL levels (current study), while at the same time enhancing mitochondrial function and proteostasis.^12−14^ These effects may hold also significant therapeutic potential for Hereditary Sensory Neuropathy Type 1 (HSN1), a rare inherited peripheral neuropathy characterized by the progressive degeneration of sensory and autonomic neurons driven by the toxic accumulation of deoxy-SLs.^27^ In combination, these observations hence warrant further evaluation of the efficacy of pharmacological SPT inhibition across this diverse spectrum of conditions.

## Materials and Methods

### Mouse Handling

Young (2 month-old) and aged (18 month-old) male C57BL/6JRj mice were purchased from Janvier Laboratories. Housing conditions involved maintaining two to five animals per cage under a 12 h light/dark cycle, with continuous access to food and water. Animals were fed a standard chow diet (Safe 150) with the following composition: 56.9% nitrogen-free extract, of which 41.0% was starch and 3.4% was sugars, 18.0% crude protein, 4.8% crude fat, 4.2% crude ash, 4.1% crude fiber and 12.0% moisture. Young (2 month-old) and old (18 month-old) mice were monitored for 20 weeks. Mice were administered either DMSO as a control or ALT-007. ALT-007 was dissolved in DMSO or DMSO alone were mixed in the food (1 mg/kg for ALT-007 or equivalent volume of DMSO for controls). Body weight and food intake measurements were recorded weekly until sacrifice.

In vivo phenotyping tests commenced after 7 weeks of diet (at 15 weeks of age for young mice and at 79 weeks of age for old mice) and were conducted every 1–2 weeks, following the outlined pipeline in Figure 2A, with the aim of minimizing stress on the animals. All animal experiments adhered to Swiss ethical guidelines and received approval from the Service de la Consommation et des Affaires Vétérinaires of the Canton de Vaud (license VD3341 × 1).

### Sphingolipid and Deoxysphingolipid Analysis

Sphingolipid and deoxysphingolipid measurements were performed by liquid chromatography–tandem mass spectrometry (LC–MS/MS) analysis as described in previous studies.^12,13,28−30^ First, cell pellets (∼1.2 × 106 cells) and muscle tissue (20 ± 5 mg, gastrocnemius muscle) were lysed by the addition of 100 and 200 μL of methanol (100%), respectively, spiked with the stable isotope-labeled internal standards (Spa(d17:0), Cer(d18:1/16:0)-d9, Cer(d18:1/18:0)-d7, Cer(d18:1/24:0)-d7 and Cer(d18:1/24:1)-d7). The Cryolys Precellys Tissue Homogenizer with ceramic beads homogenized the samples by two 20 s rounds at 10.000 rpm (Bertin Technologies) with air-cooled bead beating at a flow rate of 110 L min-1 and 6 bar.

The homogenized extracts were centrifuged (15 min at 4,000*g*, 4 °C) and the resulting supernatants were collected for LC–MS/MS analysis. LC–MS/MS was performed in positive ionization mode using a 6495 triple-quadrupole system coupled to a 1290 ultrahigh performance liquid chromatography (UHPLC) system (Agilent Technologies), adapted from previously described methods.^31^ The chromatographic separation was performed on a ZORBAX Eclipse Plus C8 column (Agilent) (1.8 μm, 100 × 2.1 mm inner diameter). Mobile phase A consisted of 5 mM ammonium formate and 0.2% formic acid in H2O and mobile B consisted of 5 mM ammonium formate and 0.2% formic acid in methanol, at a flow rate of 400 μL min-1 was used. The sample injection volume was 2 μL, and the column temperature was maintained at 40 °C. A linear gradient was applied and held until minute 14, with elution starting from 80% to 100% of B in 8 min. The column was then equilibrated to the initial conditions.

ESI source conditions were used as follows: dry gas temperature, 230 °C; nebulizer, 35 psi and flow rate of 14 L min^–1^; sheath gas temperature, 400 °C and flow rate of 12 L min^–1^; nozzle voltage, 500 V; and capillary voltage, 4000 V. Dynamic multiple reaction monitoring was used as the acquisition mode with a total cycle time of 500 ms. Optimized collision energies for each metabolite were used. Raw LC–MS/MS data were processed applying Agilent Quantitative Analysis software (version B.07.00, MassHunter Agilent Technologies).

Absolute quantification was based on calibration curves and stable isotope-labeled internal standards to determine the response factor for each metabolite, with linearity assessed using 12-point range standard curves. Peak area integration was subject to manual curation and correction where necessary. Sphingolipid concentrations were reported and protein concentrations in protein pellets (measured by BCA, Thermo Fisher Scientific) were determined after metabolite extraction. Theoretical transitions for 15 dihydrosphingomyelin species were added using the in silico MS/MS LipidBlast database, and retention times were predicted based on similarities to other sphingolipid species targeted by the same analytical method. Semiquantification was performed by using integrated SRM peak areas.

### Body Composition Analysis (Echo-MRI)

At the first and last (week 20) after experiment onset (as indicated in Figure 2A), body composition analysis was conducted. Each mouse was placed for short in an Echo-MRI (magnetic resonance imaging) machine, specifically the 3-in-1 model from Echo Medical Systems. This procedure recorded lean mass, along with total body weight, with each measurement taking approximately 1 min per individual.

### Hanging Test

Mice were allowed to acclimate to the testing room for 30 min before the experiment. Each mouse was positioned on a wire grid, gripping it with all four limbs. The grid was then inverted, causing the mouse to hang, and the duration it could maintain this position was recorded. The maximum trial length was set at 2 min and 30 s (150 s). The latency to fall was measured five times for each mouse, with 10 min intervals between trials. The test was performed 7 weeks post experiment onset as indicated in Figure 2A.

### Grip Strength

Grip strength was evaluated using a grip strength test. Each mouse’s grip strength was measured using a pulldown grid assembly connected to a grip strength meter (Columbus Instruments). The mouse was pulled along a straight line parallel to the grip to determine peak force. The experiment was conducted three times, and the highest recorded value was considered for the analysis. The assay was performed 8 weeks post experiment onset as indicated in Figure 2A.

### Rotarod Test

The rotarod test assesses muscle strength, coordination, and endurance.^32^ Mice were allowed to acclimate in the room for 30 min. The rotating cylinder (rotarod) speed gradually increased from 0 to 40 rpm over 5 min. Each mouse underwent three trials per day for three consecutive days. The latency at which the mouse either reached passive rotation or fell from the rotor was recorded. The results presented here focus on the latency of the best trial conducted on the second day. The test was performed 9 weeks post experiment onset as indicated in Figure 2A.

### VO2max Test

VO2max in mice was measured with a calorimetric treadmill (Columbus Instruments) with an incremental speed protocol as previously described.^33^ The assay was performed 11 weeks post experiment onset as indicated in Figure 2A.

### Uphill Running

Animals were placed on a treadmill at a speed of 15 cm/s in the beginning, then speed was increased by 3 cm/s every 12 min, with an incline of 5°. Mice were taken off the treadmill if they were considered exhausted (5 or more shocks (0.1 mA) per minute for two consecutive minutes) or if the maximum duration of the test was reached (3 h). The maximum running time before exhaustion was recorded and registered as maximum uphill running time.^34^ The test was performed 13 weeks post experiment onset as indicated in Figure 2A.

### Metabolic Cages

Energy expenditure and activity during day and night were measured using the CLAMS monitoring system (Columbus instruments). Mice were housed in metabolic cages for 48 h. Animals were placed in CLAMS cages 19 weeks post experiment onset as indicated in Figure 2A.

### Oral Glucose Tolerance Test (OGTT)

For OGTT, mice underwent an overnight fast, and on the morning of the experiment, they were administered a gavage consisting of a 20% glucose solution in water (10 mL [2 g]/kg body weight). Blood glucose levels were monitored from the tail vein using a glucometer prior to the gavage and at 15, 30, 45, 60, 90, 120, 150, and 180 min thereafter. Additionally, blood samples were collected at 0 min (pregavage), 15 and 30 min to assess fasting insulin levels and glucose-stimulated insulin secretion. oGTT was performed 17 weeks post experiment onset as indicated in Figure 2A.

### Tissue Collection

Mice were sacrificed 20 weeks after experiment onset as indicated in Figure 2A. Prior to sacrifice, mice were fasted for 4 h in the morning. Animals were sacrificed between 1:30 and 4 p.m. Mice were anesthetized with isoflurane, followed by a complete blood draw from the vena cava and perfusion with cold phosphate-buffered saline. Immediately thereafter, various tissues including liver, kidney, spleen, heart, brain, gastrocnemius muscle and tibialis anterior muscle were harvested, weighted and promptly flash-frozen in liquid nitrogen.

For blood samples, collected in EDTA-coated tubes, centrifugation was conducted at 4500 rpm (rpm) for 10 min at 4 °C. The resulting plasma supernatant was then flash-frozen in liquid nitrogen for subsequent plasma analyses. Sections of the liver and kidney were preserved in formalin or optimal cutting temperature compound for histological analysis.

### Plasma Analysis

Plasma parameters were assessed using samples diluted two times (at a 1:1 ratio of plasma to diluent) with the DimensionXpand Plus system from Siemens Healthcare Diagnostics. The biochemical tests were conducted in accordance with the manufacturer’s instructions for each specific parameter. These parameters included transaminase AST (DF41A), transaminase ALT (DF143) and creatine kinase (11097640), all provided by Siemens Healthcare.

For the measurement of plasma levels of TIMP-1, FGF-21, and GDF-15, the Mouse Premixed MultiAnalyte Kit (LXSAMSM) from R&D Systems was employed. The analyses were carried out using a Luminex 200 system, following the manufacturer’s instructions.

### Aggresome Detection

Primary myoblasts from IBM patients and healthy aged matched donors were provided by Hospices Civils de Lyon. Cells were grown in DMEM/F-10 supplemented with 12% FBS (Gibco) and penicillin–streptomycin (1×, Gibco). Cells were cultured on sterilized coverslips in six-well plates (Greiner BioOne, CELLSTAR, 657160). Fixation was performed with Fixx solution (Thermo Fisher Scientific, 990244) for 15 min, followed by permeabilization with a 0.1% Triton X-100 (Amresco, 0694) solution for an additional 15 min at 21 °C. After two washes in PBS, the cells were incubated with proteostat dye (PROTEOSTAT Aggresome detection kit, Enzo Life Science) and DAPI (Invitrogen) for 30 min at 21 °C. Slides were then washed with PBS and mounted with DAKO mounting medium (DAKO, S3023). Image acquisition for these cultured cells was performed with a 63× oil lens on a Leica SP8 confocal microscope, and subsequent image processing utilized Fiji software (version 1.47b; http://imagej.nih.gov/ij).

### RNA Seq Analysis

RNA seq analysis was performed as previously described. Changes in cell composition were assessed by gene set enrichment analysis (GSEA) with the *clusterProfiler* package (Yu G. et al., 2012) and the MsigDB v7.5.1 mouse cell type signature set, accessed through the *msigdbr* package was performed. Only the gene sets relevant to skeletal muscle and neuronal cell types were retained.

### Statistical Analysis

Differences between two groups were assessed using two-tailed *t* tests. Differences between three groups were assessed using one-way analysis of variance (ANOVA) with Tukey’s post hoc testing. Variability in all plots and graphs is presented as the SEM *P <* 0.05 was considered to be statistically significant. *P < 0.05, **P < 0.01, and ***P < 0.001. GraphPad Prism 10 (Prism) was used for statistical analyses.

### *C. elegans* Strains

Strains used were wild-type Bristol N2, GMC-101 (unc-54p::A-beta-1–42::unc-54 3′-UTR + mtl-2p:GFP), CL2122 (dvIs15 [(pPD30.38) unc-54(vector) + (pCL26) mtl-2::GFP]) and CL2120 (dvIs14 [(pCL12) unc-54::beta 1–42 + (pCL26) mtl-2::GFP]) and were provided by the Caenorhabditis Genetics Center (University of Minnesota).

### Lifespan Measurements in *C. elegans*

Lifespan assays were conducted at 20 °C unless otherwise noted. Synchronous populations were generated by hypochlorite treatment of gravid adults to obtain tightly synchronized embryos, which were then allowed to develop into adulthood under defined conditions. 20–25 worms were placed on NGM plates containing 5-FU seeded with *E. coli* OP50 bacteria. ALT-007 at a final concentration of 5 μM was added to the top of the plates diluted in DMSO, and DMSO was used at the same concentration as a control. Each condition included at least 170 animals per experiment.

The day of egg collection was designated as *t* = 0. Animals were transferred to fresh plates every 3 days and examined daily for touch-provoked movement and pharyngeal pumping, until death. Worms that died due to internal hatching of eggs, extruded gonads, or desiccation from crawling off the plate were censored accordingly. Each survival assay was repeated at least twice, and figures show representative results.

Survival curves were generated using the Kaplan–Meier product-limit method. The log-rank (Mantel–Cox) test was used to assess differences in survival and calculate *P* values. Statistical analyses and lifespan values were determined using GraphPad Prism 10 software. Complete lifespan statistics are presented in Supporting Information Table 1.

### Paralysis Scoring

At least 100 worms per condition were manually scored for paralysis after poking. Results are representative of data obtained from at least two independent experiments. ALT-007 was administered at 1 μM and DMSO was used as a control. GMC101 animals were used and scoring was performed at day 1 of adulthood.

### Western Blot Analysis in *C. elegans*

Sample preparation: Synchronized animal populations were collected and washed in M9 buffer. After washing, two volumes of homogenization buffer (20 mM Tris, pH 7,4, 20 mM NaCl and 1 mM MgCl2) plus complete mini proteinase inhibitor cocktail (Roche) in a final concentration 1× was used. One volume of beads (0.5 mm zirconium oxide beads) was added and samples were homogenized. After homogenization 6×, Laemmli sample buffer with b-mercaptoethanol was supplemented to a final concentration of 1×. Protein samples were analyzed by Tricine-SDS–polyacrylamide gel electrophoresis (SDS–PAGE) and transferred to nitrocellulose membrane. Proteins were detected using the following antibodies under the manufacturer’s conditions: *anti*β-actin (Sigma) and antiAmyloid-beta, 1–16 (6E10) (BioLegend).

## Supporting Information Available

Plasma Citrulline levels and alanine/serine ratio in gastrocnemius muscle (Supplementary Figure 1), Gene set enrichment analysis (Supplementary Figure 2) and *C. elegans* lifespan data (Supplementary Table 1) (PDF)
