# Supplementary material for: Safe and Orally Bioavailable Inhibitor of Serine Palmitoyltransferase Improves Age-Related Sarcopenia
Source: ACS Pharmacol Transl Sci. 2024 Dec 29;8(1):203–15. doi: 10.1021/acsptsci.4c00587 (PMC11729425; doi:10.1021/acsptsci.4c00587)
Supplement: Supplementary file 1 — pt4c00587_si_001.pdf [file pt4c00587_si_001.pdf]

Supplementary Information

A safe and orally bio-available inhibitor of serine palmitoyl transferase improves age-related sarcopenia

Johanne Poisson[1], Ioannna Daskalaki [1], Vijay Potluri [2], Jean-David Morel [1], Sandra Rodriguez-Lopez [1], Alessia De Masi [1], Giorgia Benegiamo [1], Suresh Jain [2], Tanes Lima [1] *$, Johan Auwerx [1] *$.

[1] Laboratory of Integrative Systems Physiology, École Polytechnique Fédérale de Lausanne (EPFL), Lausanne 1015, Switzerland.

[2] Intonation Research Laboratories, Hyderabad 500076, India

* These two authors contributed equally to this work

$ Corresponding authors: Johan Auwerx, Tanes Lima

Email: Johan Auwerx: admin.auwerx@epfl.ch

Tanes Lima: taneslima@gmail.com

Contents of Supplementary information

1. Results

   1.1. Supplementary Figure 1

   1.2. Supplementary figure 2

   1.3. Supplementary Table 1

1. Results

1.1. Supplementary Figure 1

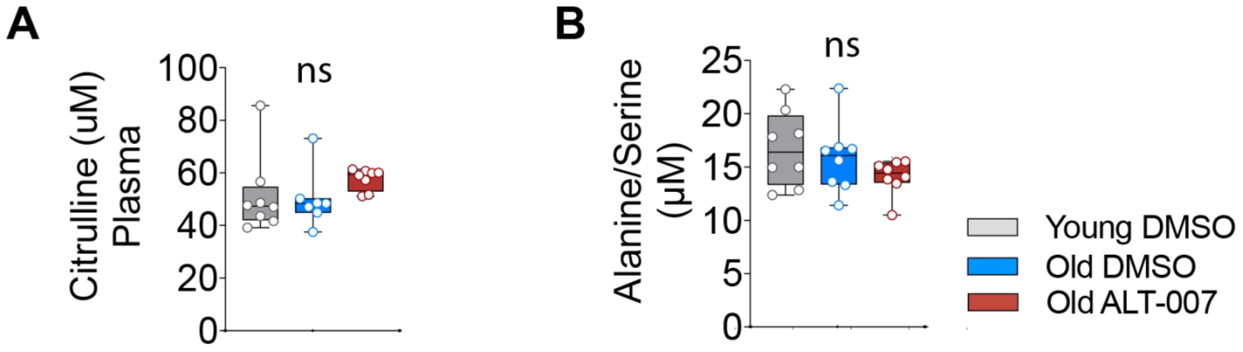

Supplementary Figure 1. ALT-007 does not affect intestinal function and alanine/serine ratio.

(A) Citrulline levels in the plasma of mice treated with ALT-007 at 1 mg/kg or vehicle DMSO (equivalent volume) admixed with the food for 20 weeks . Young mice were 28 weeks and old mice were 22 months at sacrifice.

(B) Alanine/serine ratio in gastrocnemius muscles from treated mice. Quantitative data are expressed as median with IQR and compared using the Mann-Whitney U test. All tests were 2 sided. ns, non-significant; N=8 per group.

1.2. Supplementary Figure 2

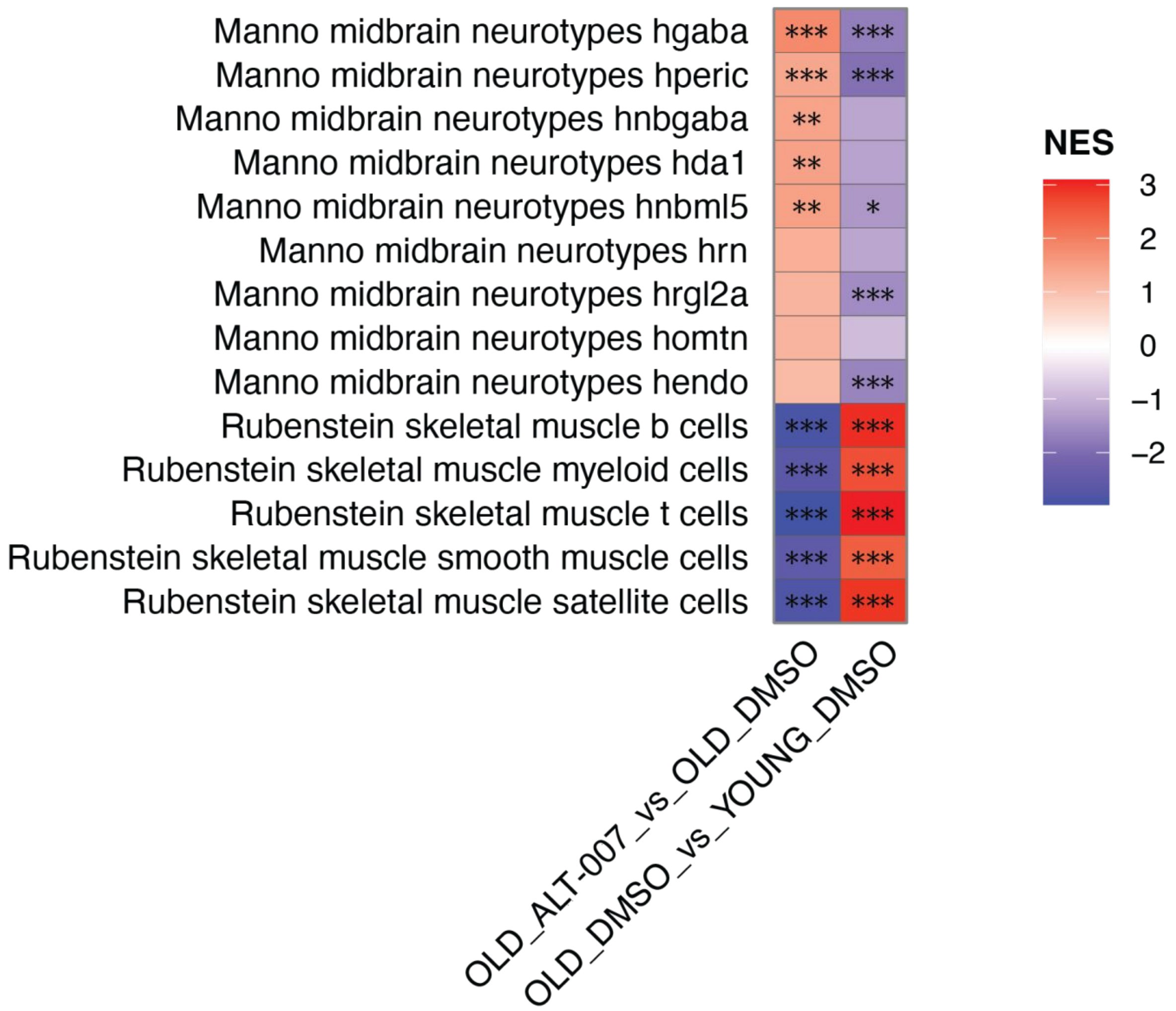

Supplementary Figure 2. ALT-007 increases neuronal cells in the skeletal muscles of old mice.

Gene set enrichment analysis using the MsigDB mouse cell type signature sets. Only the gene sets relevant to skeletal muscle and neuronal cell types were retained. GSEA was performed with the cluserProfiler package and gene sets were accessed through the MsigDBr package.

1.3. Supplementary Table 1

Suppl. Table 1. Statistics of individual lifespan experiments								
Strain	Bacteria	Compound	Concentration	Median survival (days)	Maximal survival (days)	No. of animals	P-value	exp. No
N2	OP50	DMSO	5uM	16	29	188		1
N2	OP50	ALT-007	5uM	18	35	170	<0.0001	1
N2	OP50	DMSO	5uM	15	28	208		2
N2	OP50	ALT-007	5uM	18	34	203	<0.0001	2

Supplementary Table 1. Statistics of individual lifespan experiments.
Lifespan table detailing the experimental conditions and statistical analyses for each biological replicate. The table includes information related to the experimental conditions used, the number of animals, p-values, and relevant significance testing results. Replicate 1 is highlighted in blue, while Replicate 2 is shaded in gray.
